# Perceptions of Physical Therapists of Online Introduction and Training in the Home-based Older Persons Upstreaming Prevention Physical Therapy (HOP-UP-PT) Program: A Qualitative Study

**DOI:** 10.7759/cureus.30350

**Published:** 2022-10-16

**Authors:** Christopher M Wilson, Sara K Arena, Lori Boright, Breana Knust, Aaron Krueger, Erica Wilson, Allison Zornow

**Affiliations:** 1 Rehabilitation Services, Beaumont Health, Troy, USA; 2 Physical Therapy, Oakland University, Rochester, USA; 3 Physical Therapy, Oakland Univeristy, Rochester, USA

**Keywords:** public health, fall prevention, development, focus groups, older adults

## Abstract

Introduction

Traditionally, physical therapy has adopted a tertiary approach to preventative care. However, recent trends in fall-related injuries and deaths among older individuals suggest a dire need for earlier intervention. The Home-based Older Persons Upstreaming Prevention Physical Therapy (HOP-UP-PT) program has been developed to improve the health and overall function of community-dwelling older adults at risk of functional decline. As demand continually rises for HOP-UP-PT services, online training modules have been developed to safely and efficiently provide HOP-UP-PT competency to physical therapists. The purpose of this study was to examine self-reported experiences and perceptions of physical therapists after completing an asynchronous training program to deliver HOP-UP-PT.

Methods

After securing Oakland University IRB approval, a qualitative study using a sample of convenience used two structured focus group interviews. Inclusion criteria required participants to be licensed physical therapists (PTs) in the state of Michigan providing at least 20 hours of direct patient care per week. Participants completed eight 30-minute training modules, each with a corresponding quiz. Upon completion, PTs attended one of two video conference focus groups. Data was analyzed using the constant comparative method to develop themes and concepts based on responses about the training modules and the overall HOP-UP-PT program.

Results

Twelve PTs with a median age of 31-40 years participated. Analysis of two focus group sessions identified three concepts (Novel Approach to Physical Therapy Care, Integration of a Preventative Approach into Clinical Practice, and Knowledge Translation) and ten themes (Addressing an Unmet Need, Establishing a Working Relationship with Community Centers, Applicability to Various Settings, Shifting the Mindset to a Prevention-focused Paradigm, Applicability to Physical Therapists that Care for Older Adults, Patient Engagement and Prevention, Value for the Professional, Importance of Availability of Options in a Learning Platform, Ongoing Availability of Program Resources and Tools, and Clinical Application Practice).

Conclusion

PTs identified the HOP-UP-PT program as a novel, clinically applicable, and adding value to the profession. Furthermore, its upstream focus aligns with the growing role of preventative care by PTs; however, as HOP-UP-PT is not a traditional approach, additional training and clinical support materials may facilitate adoption and clinical application. HOP-UP-PT uses a preventative approach to clinical practice, but efforts to translate knowledge to PT are an important consideration. Additionally, the study identified a need for refinement and modifications to the existing HOP-UP-PT training modules.

## Introduction

Traditionally, physical therapists (PTs) have used a restorative or tertiary prevention approach to deliver patient care versus that of a primary or secondary preventative focus. However, recent trends in fall-related injuries and deaths among older individuals suggest a substantial need for earlier intervention to thwart potential fall events. Specifically, one in adults aged 65 years and older fall each year and account for over 3 million emergency visits and $50.6 billion in medical costs annually [1]. Long-term consequences of a fall event may include financial burdens, functional decline, and/or fear of falling leading to anxiety and decreased participation in their community. In other cases, falls may result in death. Increased fall volumes are projected to rise given the aging US population. It is anticipated that fall rates among the aging population may result in up to seven deaths per hour in the United States [2,3].

One promising solution to this national crisis is founded on the idea of prevention-focused programming led by PTs. HOP-UP-PT, also known as Home-based Older Persons Upstreaming Prevention Physical Therapy, is one such program. The initiative consists of a growing network of PTs aiming to improve the health and overall function of community-dwelling older adults at risk of future functional decline. Older adults that participate in HOP-UP-PT receive individualized treatment from specially trained and certified PTs consisting of six in-person and three telehealth sessions over a seven-month period to improve safety in the home and community. In a randomized controlled trial, HOP-UP-PT participants had an eight-fold reduction in falls among those at the highest fall risk. In addition, there is evidence of a reduction in multiple fall risk factors and improved positive health behaviors in older persons from the HOP-UP-PT program [4-6]. The multifactorial program also offers an opportunity for prolific cost savings and addresses social determinants of health when broadly available to older adults [7]. Previously, PTs have received training and certification in the HOP-UP-PT model of care in person. However, in light of the COVID-19 pandemic combined with a rise in demand for HOP-UP-PT services, asynchronous delivery of the training was warranted.

In summary, the concept of prevention-focused, home-based PT services is relatively novel. Because the HOP-UP-PT program utilizes non-traditional referral sourcing and clinical approaches, a study examining perceptions of PTs as it relates to participating in training for the overall care philosophy of prevention-focused PT services and administration of the HOP-UP-PT clinical protocol was justified. The purpose of this study was to examine self-reported experiences and perceptions of PTs after completing an asynchronous training program to administer HOP-UP-PT.

## Materials and methods

Research design

After securing approval for this study from the Oakland University's institutional review board (FY2021-222), a qualitative research design was initiated with the intent of garnering feedback related to PTs’ experiences when interacting with the HOP-UP-PT’s online training modules to become a certified HOP-UP-PT provider.

Content development

**Figure 1:**
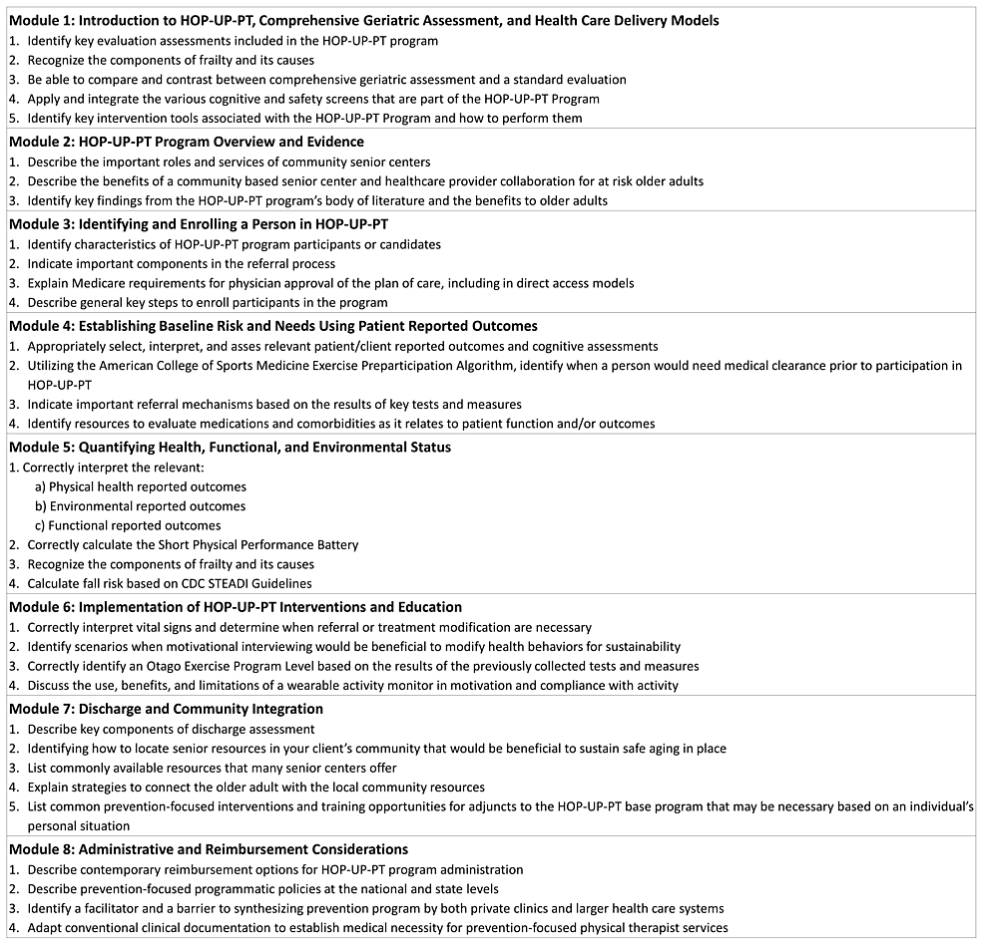
Module titles and learning objectives HOP-UP-PT, Home-based Older Persons Upstreaming Prevention Physical Therapy; CDC, Center for Disease Control and Prevention; STEADI, Stopping Elderly Accidents, Deaths, and Injuries

Prior to the initiation of the study, the training modules were created using prior in-person delivery materials as a reference. Educational materials were developed by three HOP-UP-PT content experts (CW, SA, LB) with formal training in delivering asynchronous online learning content. After initial development, the scripting, audio, and presentation formatting were vetted for face validity, context, and recording quality to ready the material for distribution by four additional members of the research team. The information was organized into eight 30-minute training modules (module titles and learning objectives detailed in Figure 1). Each of the eight modules consisted of narrated PowerPoint presentations and a streaming video of the same content posted to a private YouTube channel. Multiple choice questions were developed that correlated with the objectives of each module. Once all modules and quizzes were completed, investigators performed a thorough pilot of the content to provide insight into improvements that could be made prior to the launch of the online learning platform.

Sampling criteria

Twelve licensed PT participants were recruited using a sample of convenience in October of 2021 at the American Physical Therapy Association - Michigan Chapter Fall Research Conference held in Detroit, MI, and from professional contacts of the investigators. To participate in the online training and subsequent focus group discussions, participants met the following inclusion criteria; 1) Licensed PT in the state of Michigan 2) Over the age of 23, and 3) acknowledged that they provided direct patient PT care an average of 20 hours or more per week. Potential study participants were excluded if they did not meet the inclusion criteria. 

Methods and procedures

After securing oral and written consent, participants were provided access to the online course entitled Foundational Concepts of the HOP-UP-PT Program. The education delivery mode was designed to be asynchronous, self-paced, and available in varied formats such as narrated PowerPoint presentations, PowerPoints with transcription of voice-over in the comments section, and YouTube videos to meet learning preferences. Despite the delivery mechanism, all content was identical for each module.

After completion of eight consecutively sequenced modules, participants were required to take a quiz and receive an 80% passing score prior to moving on to the next module. Scoring less than 80% required the participant to review that module’s material and retake the quiz until a score of 80% was achieved. Upon successful completion of all eight modules, an optional, five-question survey to garner learners’ perceptions for module improvements and refinement was offered. At the completion of the course, participants were asked to fill out an anonymous survey with demographic information including age, area of practice, years of experience, and race. 

Once all 12 PTs completed all eight modules of the educational course, learners self-selected one of two synchronous online focus group meetings. These meetings utilized identical guiding questions to gain insight into perceptions and experiences with the HOP-UP-PT training modules.

Prior to the planned focus groups, an interview script was developed. The scripting and associated questions were designed using open-ended questions organized from general to specific, avoiding repetition among questions, and avoiding an excessive number of questions [8-10]. Table 1 details the 12 questions that were utilized after three content experts (authors CW, SA, LB) reviewed the interview script and questions for clarity, content, and feasibility. 

**Table 1:** Focus group interview questions

Question number	Question phrasing
1	What were your first impressions of this course as you started the training modules?
2	What are some of the strengths you think should be retained in the modules moving forward?
3	What are some improvements that you would suggest be made?
4	Would you recommend this course to your colleagues? Why or why not?
5	Did you encounter any problems or issues when completing this course? If yes, please describe.
6	Is there any information that you think would be helpful to add to this course to benefit future participants? If yes, please describe.
7	What aspects of this course helped you to learn the material best?
8	Are there any aspects of this course that were a deterrent to your learning?
9	Do you feel you have the majority of the information you need to administer the HOP-UP-PT program to an older adult?
10	Are there any other treatment strategies that you implement with your patients that you believe would be useful to the HOP-UP-PT program? If so, please explain.
11	If you or your employer were purchasing this course, what do you believe would be an appropriate price point?
12	Is there anything we have not touched on today that you would like us to know?

Six days prior to each of the two 90-minute focus group meetings, participants were notified of the meeting instructions, given the Zoom link via an email, and provided with the twelve questions planned to guide the discussion. Both focus groups occurred during the evening of the second week of December 2021. Each focus group was divided to include six participants, two primary facilitators (BK and AK, EW and AZ), and one senior investigator (LB). In order to avoid bias in responses, the primary facilitators had no relationships with participants. At the beginning of each focus group, LB introduced herself, provided information on the study, and explained the procedure (e.g., changing their displayed name on the Zoom platform to an assigned number to protect their identity). The interest of LB in the topic was grounded in her clinical experience of educating therapists about the HOP-UP-PT program. A visual and audio recording of each meeting was captured through Zoom’s (Zoom Video Communications, Inc., San Jose,CA) live recording software. After completion of the focus group meeting, each of the participants was provided with a certificate of completion that could be used for renewing their PT license.

Data analysis

Transcripts from the two Zoom focus group meetings were analyzed by all seven investigators. Four phases of data analysis took place. In the first phase, the investigators ensured the accuracy of the transcripts by reviewing the audio recordings from each Zoom meeting and editing them as appropriate. In order to ensure clarity of the responses, researchers copyedited transcripts when discrepancies were identified. After the initial review investigators met to discuss their findings.

In the second phase, each investigator independently began coding the transcripts with the intent of identifying common themes and relevant supporting data corresponding to the proposed themes. Upon completion of this second phase, all investigators met to discuss and analyze similarities among the findings and major concepts were suggested. In the third phase, investigators reviewed the transcripts to assure all proposed themes, major concepts, and quotes aligned with the data. If a theme or a concept was not unanimously identified, investigators discarded or revised the analysis. In the fourth phase, all data was reviewed until saturation and multiple meetings took place among investigators to ensure that no new themes were identified. As a result, the data became saturated and no more supporting data were identified.

We aimed to establish the trustworthiness of the data using criteria that includes: credibility by using triangulation where seven investigators analyzed the data and assessed the themes until a consensus was reached; dependability by using interview scripts and providing a significant amount of detail into the research design; and, confirmability was established by using triangulation to reduce the bias of the investigators.

## Results

Participant demographics

Participants’ demographic data, areas of practice, and years of experience are shown in Table 2. There were 12 participants (n=12) in the study. 

**Table 2:** Participant demographics

Variable		N (%) (n=12)
Gender	Female	11 (91.7%)
	Male	1 (8.3%)
	Non-binary/ third gender	0 (0%)
	Prefer not to say	0 (0%)
Race/Ethnicity	Caucasian	10 (83.3%)
	Hispanic, Latino, Spanish	1 (8.3%)
	Black or African American	0 (0%)
	Asian	0 (0%)
	American Indian or Alaskan Native	0 (0%)
	Middle Eastern or North African	0 (0%)
	Other	0 (0%)
	Prefer not to say	1 (8.3%)
Age	20-30 years old	3 (25.0%)
	31-40 years old	4 (33.3%)
	41-50 years old	3 (25.0%)
	51-60 years old	0 (0%)
	61-70 years old	0 (0%)
	71+ years old	0 (0%)
	Prefer not to say	2 (16.7%)
Experience as a Therapist	0-5 years	2 (16.7%)
	6-10 years	3 (25.0%)
	11-15 years	1 (8.3%)
	16-20 years	2 (16.7%)
	21-25 years	1 (8.3%)
	26-30 years	3 (25.0%)
	31+ years	0 (0%)
Primary Physical Therapy Practice Setting	Acute Care	7 (58.3%)
	Inpatient Rehabilitation	4 (33.3%)
	Outpatient	3 (25.0%)
	Home Health Care	2 (16.7%)
	School Based	1 (8.3%)
	Other	2 (16.7%)
Percent of Patients seen who are Older than 65 in Daily Clinical Practice	0-20%	2 (16.7%)
	41-60%	2 (16.7%)
	61-80%	5 (41.7%)
	81-100%	3 (25.0%)

Analysis of the two focus group sessions identified three concepts and ten themes which are detailed in Table 3. 

**Table 3:** Themes and concepts

Concepts	Themes
Novel Approach in Physical Therapy Care Delivery	Addressing an Unmet Need
	Establishing a Working Relationship with Community Centers
	Applicability to Various Settings
	Shifting the Mindset to a Prevention Focused Paradigm
Integration of a Preventative Approach into Clinical Practice	Applicability to Physical Therapists that Care for Older Adults
	Patient Engagement and Prevention
	Value for the Professional
Knowledge Translation	Importance of Availability of Options in a Learning Platform
	Ongoing Availability of Program Resources and Tools
	Integration via Clinical Application and Practice

Concept one: a novel approach in physical therapy care delivery

The first concept that was identified is Novel Approach in Physical Therapy Care Delivery and it has four themes: addressing an unmet need, establishing a working relationship with community centers, applicability to various settings, and shifting a mindset to a prevention-focused paradigm.

Addressing an Unmet Need in Healthcare

Participants expressed that the HOP-UP-PT clinical approach addresses an unmet need in health care, specifically the needs of at-risk older adults as it can be used to fill a healthcare gap by targeting programming to this growing segment of the US population. Participants also mentioned that the increased shortage of home health care PTs may negatively be affecting access to home-based services as PTs are essential to implementing safe intervention plans for seniors in their homes:

*“I definitely have been hearing from patients that…normally we think of home care coming two to three times a week, and these companies are so understaffed and only coming like once a week”* -Participant 4, 12/14 focus group

*”...something like this could be really helpful in making sure that they are getting appropriate exercises and still building up safely when you know the therapist isn't there.”* - Participant 4, 12/14 focus group

Establishing a Working Relationship with Community Centers

Participants discussed the importance of having an ongoing relationship with community centers as it will increase community engagement and service utilization among senior centers while also increasing the amount of preventative care opportunities available to seniors. One participant discussed how affiliations within community centers can have positive outcomes in urban communities and they can be utilized to fill the gap by providing suitable access to health care for seniors in need:

*”I liked learning about how you can work with different community centers. I live in Detroit with a lot of people who don't have good access to healthcare and I just think this is such a great way to give them some access.”* - Participant 5, 12/16 focus group

Applicability to Various Settings

Participants found that the information they learned from the modules could be used in various practice settings to educate patients or family members. Participants stated the two quotes below:

*“Even though I work in the hospital, some of the stuff I learned in the modules, I can certainly suggest to the family members when they come in for the family instruction on the new information.*” - Participant 4, 12/16 focus group

*"[I liked] the idea of a smartwatch, with the acceleration and deceleration if someone falls, and I thought [of] my mother. The cost of a LifeAlert or something like that can be very expensive monthly versus…an Apple Watch…So, stuff like that was an eye opener*.” - Participant 4, 12/16 focus group

Shifting the Mindset to a Prevention Focused Paradigm

The participants expressed difficulty shifting to a preventative care mindset as the traditional care approach and associated payment methods are best established for individuals with an injury or event that precipitates the need for care requiring multiple visits per week. A preventive approach may require less frequent visits or more time between visits, but does not negate the need for a skilled approach to dosing and/or modifying care. One participant reported their struggle as follows:

*“I guess I would just have a hard time incorporating a lot of the things that I do when I'm seeing people multiple times a week… and I'm only seeing them once every month or [so].”* - Participant 5. 12/16 focus group

Table 4 outlines participants’ examples for each theme related to the concept of the novel approach in physical therapy care delivery.

**Table 4:** Examples of themes related to the novel approach in physical therapy care delivery

Themes	Examples
Addressing an Unmet Need	Preventative care for 65+
	Increased demand for care in the home
	Expands a physical therapist’s geriatric knowledge base
	Upstream prevention care may mitigate the excessive need for downstream rehabilitation services
Establishing a Working Relationship with Community Centers	Providing access to preventative healthcare can increase community engagement and service utilization among senior centers
	Community center affiliations increase access to preventive care for many in-need seniors
Applicability to Various Settings	Knowledge learned is applicable to home care, outpatient, and inpatient
	Leveraging technology as an extension of physical therapist services (remote fall monitoring/ wearable activity monitor)
	Educating family members
Shifting the Mindset to a Prevention Focused Paradigm	Traditional rehabilitation mindset may hinder early adoption of implementation
	Integration of personalization of program delivery
	Anticipation of future barriers with the implementation of appropriate intervention strategies

Concept two: integration of a preventative approach into clinical practice

A second concept was identified as the Integration of a Preventative Approach in Clinical Practice and includes three underlying themes: applicability to physical therapists that care for older adults, patient engagement and prevention, and value for the professional.

Applicability to Physical Therapists that Care for Older Adults

Participants felt that the Foundational Concepts of the HOP-UP-PT Program course was designed toward PTs predominantly working in home health care or with geriatric patients, as evidenced by these two statements made by PTs in the focus groups:

*“I [agree] with what everyone else said, as myself being an outpatient PT working in people's homes, this is definitely geared to what I do…[However,] I don't know [that] if I did acute care full-time if it would be something that I would be using.”* - Participant 5, 12/16 focus group

*“It’s not a difficult course to gain knowledge from to be certified…there are so many areas out there that are missed. With our elderly population…this is a good way to do that.”* - Participant 2, 12/16 focus group

Patient Engagement and Prevention

Most participants agreed that going into homes to deliver this preventive care along with the PT’s creativity and education would likely facilitate patient compliance. Statements that support this theme are as follows:

*“I feel like the carryover would be really low on [exercises such as knee extensions and single leg raises]...but if the patient can see the benefit up front then they are a lot more likely to buy into doing the exercises.”*- Participant 2, 12/14 focus group

*“…as the PT going in, I have some degree of creativity to try to come up with things that are actually helpful to them.”* - Participant 4, 12/14 focus group

Value to the Professional

The participants discussed the value of the course as follows:

*“You come out of this with a certificate to be a HOP-UP-PT [provider]. I feel like that would boost what you get out of the course, not just the CEUs.”* - Participant 4, 12/14 focus group

Table 5 provides participants’ examples for the themes related to the concept of Integration of a Preventative Approach into Clinical Practice.

**Table 5:** Examples of themes related to the integration of a preventative approach into clinical practice

Themes	Examples
Applicability to Physical Therapists that Care for Older Adults	A review of foundational knowledge of key concepts (Otago, etc.) is recommended prior to taking the course
	The course is most applicable to home care or geriatric therapy
	Some skills may not be applicable to certain physical therapy settings (outpatient clinics with highly athletic patients)
	Content introduced in HOP-UP-PT may improve the quality of care when working with older adults
Patient Engagement and Prevention	Tailoring the program to address individuals’ impairments necessitates skilled physical therapist services
	Identified opportunities for patient-specific or condition-specific add-on modules
	Patient-physical therapist relationship may be enhanced in a home setting
Financial Implications	Earned credential increases inherent value of training program
	Self-paced nature of courses allows for adaptation to professional schedule
	Institutional support of HOP-UP-PT program may assist with participation or implementation
	Opportunity for additional revenue stream without a commitment to a full patient care caseload

Concept three: knowledge translation

The final concept identified in the data analysis is Knowledge Translation which has the following three themes: the importance of the availability of options in a learning platform, the ongoing availability of program resources and tools, and integration via clinical application and practice.

Importance of Availability of Options in a Learning Platform

Participants reported multiple methods of learning are essential for an online learning platform as individuals have different learning preferences. Two comments that support this theme are below:

*“I really liked the voiceovers, but [I] kept the…additional comment box open when I was viewing all of them. I liked how there are basically three [ways] that you can gather information.*” - Participant 4, 12/16 focus group

*“It was really nice that it rotated through the different presenters, and they each had their own sort of different way of presenting it and to me, that helped make each one feel kind of fresh.”* - Participant 3, 12/16 focus group

Ongoing Availability of Program Resources and Tools

Participants described the desire for a reference sheet and additional resources to aid in understanding the broader scope of the HOP-UP-PT interventional programming. Specifically, one participant stated:

*“I wish I had… the whole step-by-step [process for] HOP-UP just for me to reference on the side of the modules...instead of having to go through all the slides to find what you're looking for.”* -Participant 2, 12/14 focus group.

Integration via Clinical Application and Practice

Some participants anticipated some difficulty in translating the new content and approach into their current clinical practice paradigm. Additional material was suggested by focus group participants and included the incorporation of case studies and mentoring opportunities:

*“...it's helpful to have [a] few examples…to problem solve for different scenarios to… repeat the whole sequence or at least part of the sequence… I would want to run through it a lot before I actually went in and had a real patient do this.”* - Participant 3, 12/14 focus group

*“Let's go to Zoom for some of these new therapists that are going out into the home for the first time or so….to just describe some of the issues… [it] might help ease some of that tension…”* - Participant 2, 12/16 focus group

Table 6 provides participants’ examples for the themes related to knowledge translation. 

**Table 6:** Examples of themes related to knowledge translation

Themes	Examples
Importance of Availability of Options in a Learning Platform	Flexibility with educational delivery is desired to accommodate learning preferences/experiences
	Visual aids (flowcharts, graphs) may facilitate the learning process
	Efforts to keep participants actively engaged is necessary
Ongoing Availability of Program Resources and Tools	Development of flowsheets, and reference guide would facilitate confidence in initiating clinical application
	One-on-one mentor session prior to first patient visit could improve confidence
	Online discussion or engagement with peers and mentors could serve as an opportunity to refine understanding
Integration via Clinical Application and Practice	Introducing case study/studies in modules would improve transition to clinical practice
	Simulation or practice opportunities will assist solidifying clinical approach
	Areas of opportunity to increase efficiency during initial implementation

## Discussion

The purpose of this study was to examine self-reported experiences and perceptions of physical therapists after completing an asynchronous training program to deliver HOP-UP-PT. Preventative care is a promising approach available to PTs to use that has not achieved widespread acceptance within PT clinical practice, therefore, it warrants additional training. Within the Essential Competencies in the Care of Older Adults from the American Physical Therapy Association, prevention and wellness behaviors are included within a PT’s scope of practice [11]. Emerging evidence in populations of varied ages suggests PTs are not consistently incorporating prevention-focused interventions into the routine care of their patients [12,13]. Notably, many studies examining this approach to PT care suggest that the larger community does not recognize the potential role of PTs in preventative care. This suggests that the PT’s skills and knowledge in this area are underutilized and that there is a need for targeted educational approaches to address this gap in understanding. HOP-UP-PT was created as a preventative method for reducing functional decline and falls in community-dwelling older adults. Therefore, the HOP-UP-PT program may have a crucial role in expanding preventative care models, increasing the awareness of the wellness and health promotion role of the PT, and ultimately proving to assist in broadly reducing healthcare costs nationwide.

The engaging, self-paced learning of the HOP-UP-PT training modules in the Foundational Concepts of the HOP-UP-PT Program course was positively received by the study participants. While prior iterations of the training have been conducted in person, an adapted asynchronous format is now available. Since the onset of the COVID-19 pandemic, physical therapy education has had to transition to online synchronous, asynchronous, and hybrid formatting, similar to the delivery modes of the educational modules utilized in this study [14]. PTs’ feelings regarding the transition from a physical therapy professional education program to fully online learning during the COVID-19 pandemic have been previously reported and demonstrated that students preferred face-to-face learning [14]. Similar to the findings in the current study, PT students recommended having supplemental videos and in-person activities as complementary support services to online instruction [15]. Specifically, the current study identified that participants learning new material using an asynchronous virtual format also desired an additional face-to-face instruction component to increase their confidence prior to employing the program in their clinical practice. One option to improve the preparedness of participants is to include a hybrid learning opportunity where the participants have the chance to talk to experienced HOP-UP-PT practitioners, either via web conference or in person. This will give the participants the opportunity to ask questions, clarify information to refine their understanding, and network with HOP-UP-PT mentors. By working with these mentors, participants will have more knowledge and feel more prepared when they start providing care to individuals in the HOP-UP-PT program. Other participants voiced the importance of including case studies in the modules to apply the skills learned and having a master pocket guide or clinician handbook to refer to when practicing in the clinic. Ultimately, participants expressed the need and desire for additional training to become confident HOP-UP-PT practitioners, which has since been included in the HOP-UP-PT training modules.

Limitations

Limitations to the current study included potential omissions or incorrect transcription when utilizing a platform-based transcription service and the potential for participant internet connectivity disruptions. Additionally, 12 participants from one geographical region and a majority who identified as female limit the generalizability of the findings. Specifically, this study had a lower male representation of participants than the 35% of male PTs in practice [14]. This suggests future research should be mindful of the geographical and demographic distributions of the participant pool. 

Future research

The perspectives of PTs working in diverse populations would be beneficial for content refinement and for obtaining the goal of a nationwide program, including barriers for PTs in communities with decreased availability of safe community centers or geographical divides (e.g., rural communities). Another option for future research would be to conduct a similar project or observational study after developing and incorporating more case studies and resource materials into the modules. Also, surveying the PTs after they have conducted a visit or the whole HOP-UP-PT program to get their perspectives after they have used their training with patients would be of interest.

## Conclusions

This study described the perceptions of PTs on eight training modules for the HOP-UP-PT program. It revealed that participants desired thorough mentoring and written support materials, which have since been developed as a result of this study. Overall, the study showed the benefits of the training modules for physical therapists that want to participate in the HOP-UP-PT program, including those that work in certain settings, such as acute or home care. Participants felt that HOP-UP-PT was a novel approach to physical therapy care that integrated a preventative approach into clinical practice, however, efforts toward improved knowledge translation into practice are important. The results of this study will help enhance the training process for therapists in the future to prepare them for working with patients in the home as part of the HOP-UP-PT program.

## References

[1] (2022). Older adults. https://health.gov/healthypeople/objectives-and-data/browse-objectives/older-adults.

[2] (2022). Older adult fall prevention. https://www.cdc.gov/falls/facts.html.

[3] (2022). Projected future growth of older population. https://acl.gov/aging-and-disability-in-america/data-and-research/projected-future-growth-older-population.

[4] Naccarato A, Wilson CM, Arena SK (2021). Perceptions of rehabilitation managers on implementation of the home-based older person upstreaming prevention (HOP-UP) program: a retrospective qualitative analysis. Cureus.

[5] Wilson C, Arena SK, Starceski R, Swanson K (2020). Older adults' outcomes and perceptions after participating in the HOP-UP-PT program: a prospective descriptive study. Home Healthc Now.

[6] Arena SK, Wilson CM, Boright L, Peterson E (2021). Impact of the HOP-UP-PT program on older adults at risk to fall: a randomized controlled trial. BMC Geriatr.

[7] Wilson CM, Arena SK, Deel C, Flasher E, Romolino N, Morris E, Boright LE (2022). Implementing home-based prevention physical therapy: a scoping review and path to launch of HOP-UP-PT. Home Healthc Now.

[8] Masadeh M (2012). Focus group: reviews and practices. Int J Appl Sci Technol.

[9] Danner MJE, Pickering JW, Paredes TM (2018). Using focus groups to listen, learn, and lead in higher education. https://styluspub.presswarehouse.com/browse/book/9781620365977/Using-Focus-Groups-to-Listen-Learn-and-Lead-in-Higher-Education.

[10] Stewart DW, Shamdasan PN (2014). Focus groups theory and practice. https://us.sagepub.com/en-us/nam/focus-groups/book239690.

[11] Wong R, Avers D, Barr J, Ciolek C, Klima D, Thompson M (2011). Essential competencies in the care of older adults at the completion of the entry-level
physical therapist professional program of study. https://aptageriatrics.org/wp-content/uploads/2022/01/APTA_Geriatrics-PT-Essential-Competencies.pdf.

[12] Schlessman AM, Martin K, Ritzline PD, Petrosino CL (2011). The role of physical therapists in pediatric health promotion and obesity prevention: comparison of attitudes. Pediatr Phys Ther.

[13] Rethorn ZD, Covington JK, Cook CE, Bezner JR (2021). Physical activity promotion attitudes and practices among outpatient physical therapists: results of a national survey. J Geriatr Phys Ther.

[14] Ng L, Seow KC, MacDonald L (2021). eLearning in physical therapy: lessons learned from transitioning a professional education program to full eLearning during the COVID-19 pandemic. Phys Ther.

[15] (2022). American Physical Therapy Association workforce analysis data. https://www.apta.org/contentassets/5997bfa5c8504df789fe4f1c01a717eb/apta-workforce-analysis-2020.pdf.

